# DATA AND INITIAL EVIDENCE: OCCUPATIONAL CHARACTERISTICS AND LATER-LIFE PHYSICAL AND COGNITIVE HEALTH

**DOI:** 10.1093/geroni/igad104.0434

**Published:** 2023-12-21

**Authors:** Dawn Carr

## Abstract

Research on aging is often based on evaluations of health and wellbeing once individuals have already reached later life. However, many of the problems observed are based on experiences that occurred during earlier phases of life. Adults spend approximately 1/3 of their time engaged in paid work, and consequently, occupational exposures are likely to play a critical role in shaping age-related changes over time. Occupational exposures are difficult to study because most studies only collect data on work environments at one point in time or only during the latest phases of one’s career. Further, much of the data on occupations is based on subjective experiences, or in association with a small subset of occupational categories. A linkage between the Health and Retirement Study Life History Module Survey (LHMS) and the recent introduction of a new data source measuring occupational characteristics based on the Department of Labor’s O*NET data now make it possible to comprehensively evaluate full life histories of work. This symposium provides an overview of this new dataset and how it was created, and provides initial evidence of the important role that occupational histories play in shaping early departure from work due to disability, mid-life metabolic disease risks, and later life cognitive function.

